# The relationship between age related changes in strength and fitness with body size, shape and composition

**DOI:** 10.1038/s41598-025-93828-2

**Published:** 2025-03-21

**Authors:** Sophie Schulte, Till Ittermann, Stefan Gross, Ralf Ewert, Marcello R. P. Markus, Mats Wiese, Sabine Kaczmarek, Nele Friedrich, Marcus Dörr, Martin Bahls

**Affiliations:** 1 Department of Internal Medicine B, University Medicine Greifswald, Ferdinand-Sauerbruch-Str., 17475 Greifswald, Germany; 2 German Centre for Cardiovascular Research (DZHK), Partner-site Greifswald, Greifswald, Germany; 3 Institute for Community Medicine SHIP-KEF, University Medicine Greifswald, Greifswald, Germany; 4 Department of Internal Medicine A, University Medicine Greifswald, Greifswald, Germany; 5 Institute of Clinical Chemistry and Laboratory Medicine, University Medicine Greifswald, Greifswald, Germany; 6 Department of Food · Nutrition · Facilities, University of Applied Sciences Münster, Münster, Germany

**Keywords:** Cardiorespiratory fitness, Hand grip strength, Anthropometric data, Longitudinal study, Observational, Biomarkers, Medical research

## Abstract

Handgrip strength (HGS), cardiorespiratory fitness (CRF) and body size, shape, and composition are all related to cardiometabolic health and are associated in cross-sectional settings. Their longitudinal relationship is less clear. We used observational data from the Study of Health in Pomerania at baseline (SHIP-TREND-0; 2008–2012) and follow-up (SHIP-TREND-1; 2016–2019) with 1,214 men and 1,293 women. HGS was measured with a hand dynamometer. CRF was assessed using cardiopulmonary exercise testing. Linear regression models were adjusted appropriately. Several sensitivity analyses were performed. From baseline to follow-up (7 years) HGS decreased in men (3.5 kg) and women (0.8 kg). VO_2_peak lessened in men (36 ml/min) and increased in women (53 ml/min). We only found significant relations in men where a 1 l decline in VO_2_peak was associated with a 0.87 kg larger decrease in fat free mass and with a 1.15 kg stronger decline in body weight. All other analysis revealed non-significant findings. This longitudinal analysis suggests that age related changes in strength and CRF are not related to body size and shape but only composition (in men). A novelty of our findings are the sex-specific aspects given that strength decreased much stronger in men compared to women.

**Supplementary Information:**

The online version contains supplementary material available at 10.1038/s41598-025-93828-2.

## Introduction

Low handgrip strength (HGS) or cardiorespiratory fitness (CRF) are associated with a higher risk of functional impairment and all-cause mortality^1,2^. The assessment of HGS is an inexpensive and simple method to measure whole-body muscle strength^3,4^. CRF, determined by cardiopulmonary exercise testing (CPET), is a well-established method for measuring physical fitness. Throughout the lifespan humans experience reductions in HGS and CRF^5–8^.

Measures of body size, shape and composition like body mass index (BMI), body weight, waist and hip circumference as well as fat mass (FM) and fat free mass (FFM) are potential risk factors for cardiometabolic disease, cardiovascular mortality and all-cause mortality^9^. Previous studies showed sex-specific associations between these markers and adverse outcomes. Men with the same BMI and age have a higher risk of coronary artery disease and cardiovascular mortality compared to women. Although women having a higher total body fat percentage, risk for adverse outcomes is determined by body fat distribution^10^. Importantly, throughout life anthropometric markers also shift towards a higher risk with increases in BMI, FM as well as waist and hip circumference.

In cross-sectional settings HGS and CRF are strongly related to body size, shape and composition. For example, a lower HGS was related with a higher BMI and FM as well as lower FFM^11–13^. In a small study of 87 elderly Swedes FFM was a significant predictor for decline in muscle strength after five years^14^. Cross-sectional studies also reported that individuals with high CRF had less FM and lower waist circumference compared to study participants with low CRF^15^. Conversely, a higher body fat was also associated with a lower CRF^16^. Nevertheless, cross-sectional studies have inherent limitations. These include for example healthy recruitment bias, which questions the generalizability to the general population, and the Hawthorne effect, which imposes that study participants change their behavior due to their participation in the study. Cross-sectional studies also have a potential risk of reverse causation^17^. Understanding the limitations of cross-sectional studies is essential to improve our understanding of risk factors and markers for health and disease.

A way to possibly overcome the risk for reverse causation are longitudinal studies. Hence, we explored the relationship between changes in CRF and HGS in relation to body size, shape and composition in a longitudinal population-based setting. Our analysis may further elucidate how age-related changes in physical fitness, strength and body size, shape and composition are related to health and disease over time.

## Methods

### Study population

**Fig. 1:**
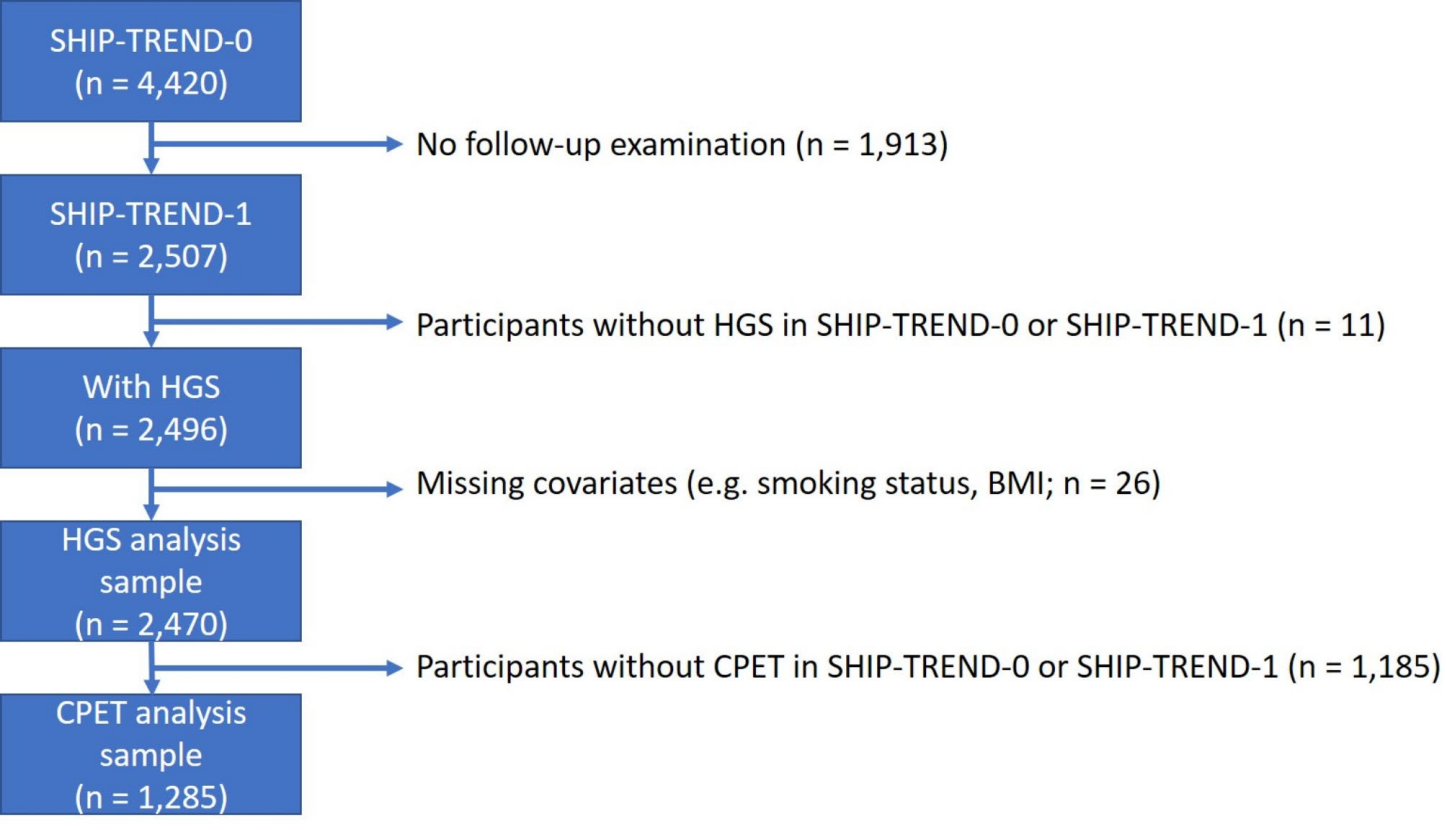
Flowchart of the included study participants in SHIP-TREND and SHIP-TREND-1. Abbreviations: BMI – body mass index, CPET – cardiopulmonary exercise testing, HGS – hand grip strength, SHIP – study of health in Pomerania.

The Study of Health in Pomerania (SHIP) is an epidemiological project in Northeast Germany. A sample was selected from the entire study population in this region based on the residents’ registration offices^18^. A two-stage cluster sampling method was adopted from the WHO MONICA (Multinational MONItoring of trends and determinants in CArdiovascular disease) project in Augsburg^19^. The study area was divided into four regions. For each region, the target population was divided into 24 age and sex groups, from which an equal number of participants were drawn. The baseline study used for this analysis is called SHIP-TREND-0. The data was collected between 2008 and 2012 with 4,420 study participants (2,145 men and 2,275 women) aged 20 to 80 years. The follow-up study is called SHIP-TREND-1 and was conducted from 2016 to 2019 with 2,507 study participants. HGS was measured for 2,496 and CRF was determined for 1,285 study participants in both cohorts (see Fig. 1). HGS was measured during the main examination while CPET was performed at a second examination day. The study is in accordance with the Declaration of Helsinki and was approved by the ethics committee of the University of Greifswald (BB 39/08 and BB 174/15). All study participants gave written informed consent. The following methods were used in the same way in both cohorts.

### Interview and physical assessments

Medical history was obtained by trained and certified personnel using a standardized computer-assisted interview. To assess physical activity (PA), study participants were classified as inactive if they did not engage in at least one hour of leisure-time PA per week in summer or winter.

Standardized measurements were used to collect anthropometric data in both cohorts. Height and weight were determined with a calibrated scale and BMI was calculated with these values (weight in kg/height² in m²). Waist circumference in cm was measured using an inelastic band at the midpoint between the lower edge of the ribs and the iliac crest in the horizontal plane of the standing study participant. Hip circumference in cm was determined as the greatest circumference between the highest point of the iliac crest and the crotch. FFM and FM were determined by bioelectrical impedance analysis (BIA) using a multifrequency Nutriguard-M device (Data Input, Pöcking, Germany) and NUTRI4 software (Data Input, Pöcking, Germany). Study participants with a pacemaker were excluded. Electrodes were placed on the hand, wrist, ankle and foot. The test frequency was 5, 50 and 100 kHz according to the manufacturer’s instruction^20^. Although differences between BIA and DEXA exist, both methods are interchangeable at the population level^21^. Further, although BIA underestimates fat-free mass, this method is useful to assess body composition^22^. For each anthropometric marker the change between SHIP-TREND-0 and SHIP-TREND-1 was calculated as change in 5 years for each participant: (Value(TREND-1) - Value(TREND-0)/Follow-Up time in years)*5.

### Hand grip strength

In SHIP-TREND-0 HGS was measured using a Smedley-type hand grip dynamometer (Scandidact, Odder, Denmark). The validity and reliability of this device has been demonstrated in several studies^23,24^. The measurement was performed once on each hand while standing. The upper arm was placed against the trunk and flexed 90 degrees at the elbow. Study participants were instructed to squeeze the handle as hard as possible for 3 s and the maximum contractile force in kilograms (kg) was recorded. The highest grip force recorded for the left and right hands was used for the present analyses.

In SHIP-TREND-1, the measurement of HGS was carried out with the Jamar Plus Digital Dynamometer (Patterson Medical, Sammons Preston, Bolingbrook, IL), which is a frequently-used HGS measuring device with high reliability and validity^25^. HGS was measured in kilograms three times for each hand. The maximum value out of six recorded measurements was used for analysis. Data of both devices were compared in 100 individuals and revealed the conversion formula HGS (Jamar device) = 0.16 + 1.04*HGS (Scandidact device), which was applied to the HGS values in TREND-0. The difference (ΔHGS) between the maximum values of SHIP-TREND-0 and SHIP-TREND-1 was calculated as change in 5 years for each participant: (HGS(TREND-1) - HGS(TREND-0)/Follow-Up time in years)*5.

### Cardio pulmonary exercise testing

Symptom-limited exercise testing was performed on a calibrated electromagnetically braked cycle ergometer with electric seat height adjustment (Ergoselect 100, Ergoline, Germany) according to a modified Jones protocol (stepwise increase in workload of 16 W/min, starting as unloaded cycling with ergometer-induced continuous exercise)^26^. The same system was used for baseline and follow-up assessments. All tests were performed at room air according to current guidelines for exercise testing, with ECG, blood pressure and oxygen saturation monitored continuously^27^. Gas exchange and respiratory variables were analysed breath-by-breath by a computerized system at 10-second intervals. Study participants were encouraged to maximize their workload before beginning the test. Peak oxygen uptake (VO_2_peak) was defined as the highest 10-second average of absolute oxygen uptake during the last minute of exercise. Exercise duration was examined from the start of exercise (excluding rest) to its completion. The difference (ΔVO_2_peak) between the values of SHIP-TREND-0 and SHIP-TREND-1 was calculated as change in 5 years for each participant: (VO_2_peak (TREND-1) - VO_2_peak (TREND-0)/Follow-Up time in years)*5.

### Statistical analysis

Multivariable regression models were used to investigate associations of ΔHGS and ΔVO_2_peak with 5-year-changes in body size, shape and composition. For this purpose, we used sex-specific linear regression models with different confounder sets. The first model was adjusted for age, baseline outcome, smoking status at baseline and at follow-up. Models with FFM were also adjusted for FM and models with FM were adjusted for FFM. The underlying reasoning is that the FFM can be increased not only by PA but also by weight gain. This means that the changes in FFM and FM cannot be considered independently of each other. Further models were calculated, which were additionally adjusted for the presence of metabolic syndrome and inactive lifestyle at baseline and follow-up or adjusted for the median of the respective outcome at baseline and follow-up instead of only the baseline outcome. In women, we calculated further models stratified by menopause status to baseline. Also, a trajectory analysis for HGS as well as VO_2_peak was performed, in which the study participants were divided into the three groups (a) increase in HGS or VO_2_peak between TREND-0 and TREND-1 (highest quartile), (b) decrease in HGS or VO_2_peak between TREND-0 and TREND-1 (lowest quartile) and (c) similar values in HGS or VO_2_peak in TREND-0 and TREND-1 (second and third quartile). Groups a and b were compared against group c. Calculations were carried out using Stata 18.0 (Stata Corporation, College Station, TX, USA).

## Results

### Study sample characteristics

The sample included 2,496 study participants (1,285 women; 51.48%) for HGS and 1,232 study participants (639 women; 51.87%) for VO_2_peak. The average follow-up time was 7.3 years. HGS, CRF and the anthropometric data are presented in Table 1 with baseline and follow-up measurements. Men were on average 50 years old at baseline and 58 years at follow-up. Women were on average 49 years old at baseline and 56 years old at follow-up. BMI, body weight and waist circumference increased in both sexes over the course of the study. Hip circumference decreased in men and increased in women. In men and women had more FM at follow-up compared to baseline. FFM, PA and the proportion of smokers decreased independent of sex. The prevalence of metabolic syndrome increased in men and women. HGS decreased in both sexes, while VO_2_peak declined in men and increased in women.

**Table 1:** Sex-stratified characteristics of the study sample.

	N men	N women	Baseline	Follow-up	*p*	Baseline	Follow-up	*p*
			Men			Women		
Age (years)	1,214	1,293	50 (40 ; 61)	58 (47 ; 68)	< 0.001	49 (39 ; 60)	56 (46 ; 67)	< 0.001
Body height (cm)	1,211	1,289	177 (173 ; 181)	177 (172 ; 181)	< 0.001	165 (160 ; 169)	164 (159 ; 169)	< 0.001
Body weight (kg)	1,213	1,288	87.3 (78.7 ; 97.2)	87.6 (78.5 ; 96.8)	0.921	70.8 (63 ; 80.4)	71.8 (63.0 ;82.3)	< 0.001
BMI (kg/m^2^)	1,211	1,288	27.9 (25.5 ; 30.5)	27.9 (25.6 ; 30.8)	< 0.001	26.1 (23.1 ; 29.69)	26.6 (23.5 ; 30.6)	< 0.001
Waist circumference (cm)	1,211	1,285	95 (88 ; 104)	102 (93 ; 109)	< 0.001	81 (74 ; 91)	90 (80 ; 101)	< 0.001
Hip circumference (cm)	1,212	1,284	101 (96 ; 106)	100 (95 ; 106)	0.130	100 (93 ; 108)	102 (95 ; 111)	< 0.001
FM (kg)	916	888	20.6 (16.3 ; 25.6)	23.8 (18.7; 29.8)	< 0.001	23.8 (18.2 ; 30.5)	26.8 (20.6 ; 34.1)	< 0.001
FFM (kg)	895	863	67.0 (61.6; 72.5)	64.3 (58.7; 69.8)	< 0.001	47.2 (44.1; 51.1)	46.2 (42.4 ; 49.9)	< 0.001
Current smokers	1,205	1,272	23.90%	19.50%	< 0.001	23.35%	19.03%	< 0.001
Physical activity	1,203	1,270	70.99%	69.24%	0.267	73.46%	70.87%	0.078
Metabolic syndrome	1,196	1,277	31.52%	32.20%	0.764	16.05%	18.94%	0.008
HGS (kg)	1,211	1,285	50.6 (44.9 ; 56.3)	47.1 (40.4 ; 53.0)	< 0.001	29.8 (26.2 ; 34.5)	29.0 (24.9 ; 33.1)	< 0.001
VO_2_peak (ml/min)	649	639	2,509 (2,149; 2,900)	2,473 (2,104; 2,880)	0.011	1,659 (1,430 ; 1,934)	1,712 (1,485 ; 1,953)	< 0.001
VO_2_peak FFM	593	593	37.2 (32.0 ; 43.5)	38.7 (32.9 ; 44.5)	< 0.001	35.6 (30.8 ; 40.2)	37.7 (32.9 ; 42.4)	< 0.001

Continuous variables are presented as median (25th and 75th percentile), categorial variables are expressed in numbers (percentages %). P-values were calculated using t-test (continuous variables) or χ2 test (categorical variables). Abbreviations: BMI – body mass index, FM – fat mass, FFM – fat free mass, HGS – hand grip strength, VO_2_peak – peak oxygen uptake, VO_2_peak FFM – peak oxygen uptake normalized for fat free mass.

### Association of ΔVO_2_peak or ΔHGS with anthropometric markers

**Fig. 2:**
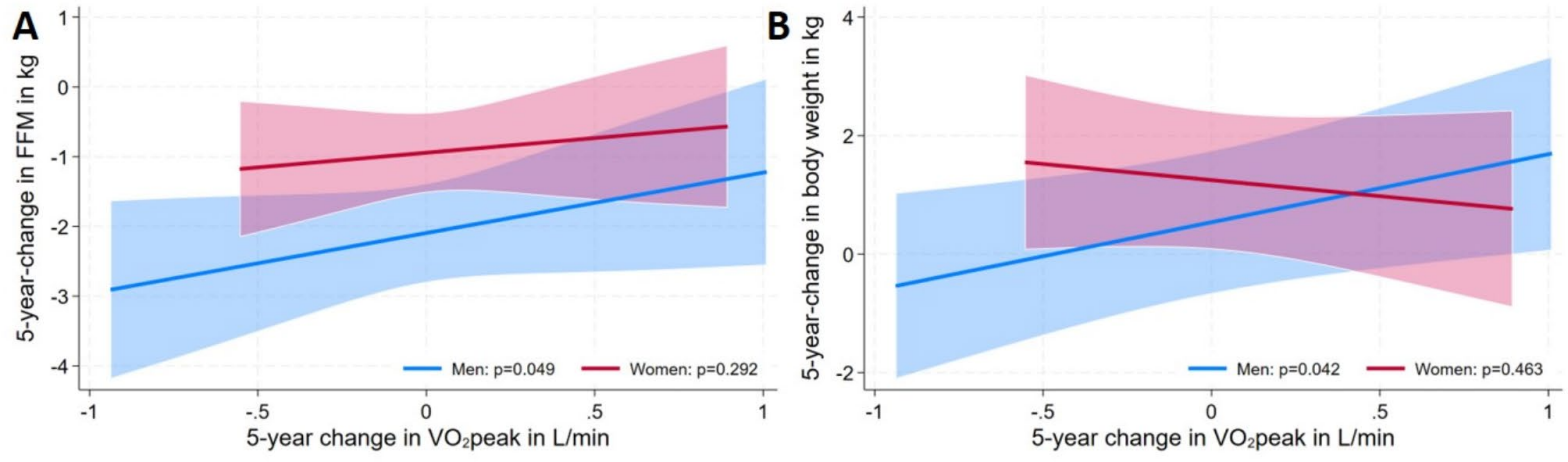
– Results for the association between changes in VO_2_peak and FFM (adjusted for Model (1) i.e. age, baseline outcome, FM, smoking baseline and follow-up) and body weight (adjusted for Model (2) i.e. PA, metabolic syndrome, age, baseline outcome, smoking baseline and follow-up). Abbreviations: FFM – fat free mass, FM – fat mass, PA – physical activity, VO2peak – peak oxygen uptake)

**Fig. 3:**
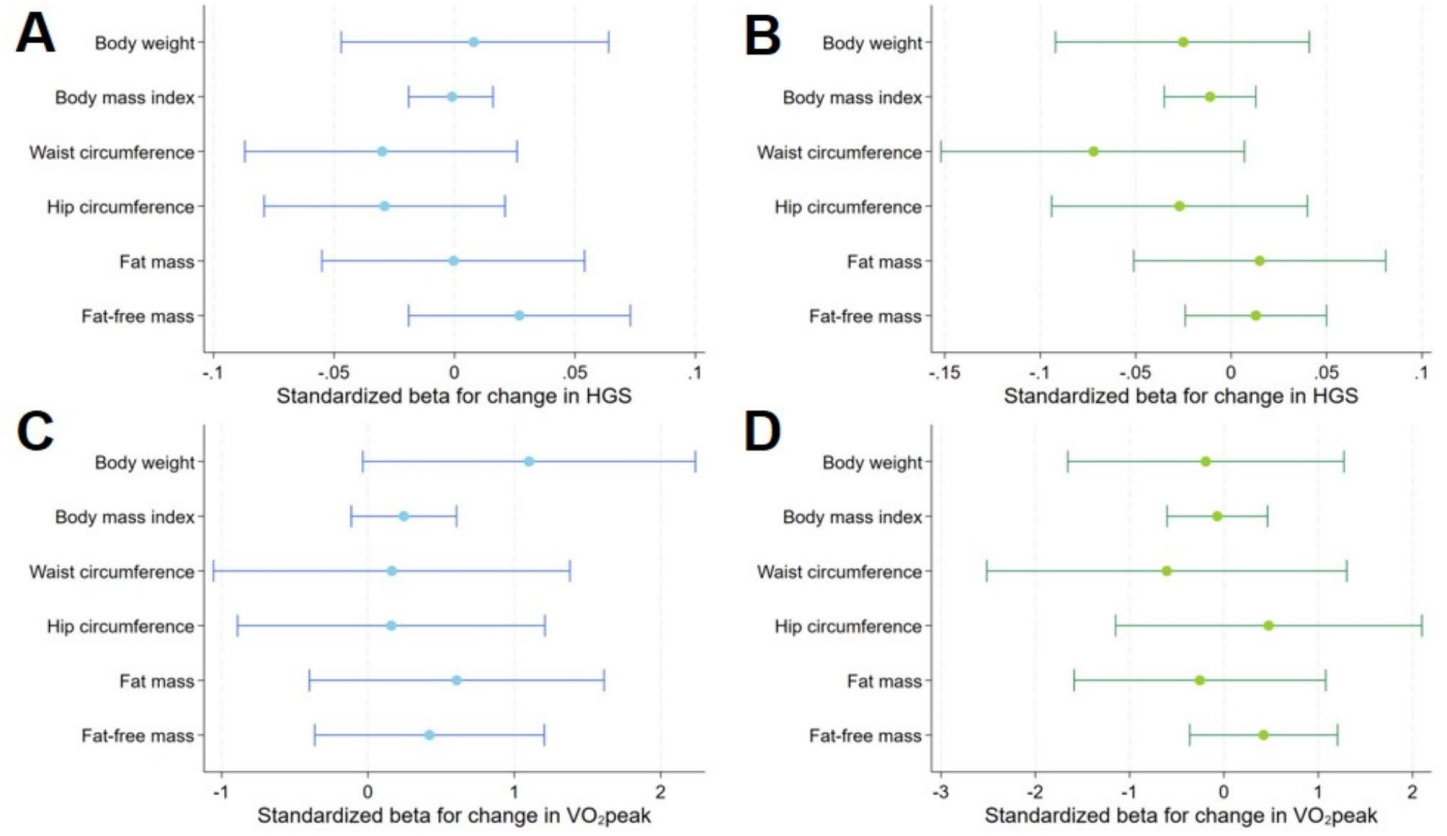
Relationship between change in weight, BMI, waist circumference, hip circumference, fat-mass and fat free mass with hand grip strength and peak oxygen uptake in men (**A**,** C**) and women (**B**,** D**). Model (1) was adjusted for age, baseline outcome, smoking baseline and follow-up. FM is additionally adjusted for FFM and vice versa. Abbreviations: BMI – body mass index, FM – fat mass, FFM – fat free mass, HGS – hand grip strength, VO_2_peak – peak oxygen uptake.

Adjusted for age, baseline outcome, and smoking status at baseline and at follow-up, a one liter lower VO_2_peak was related to a 0.87 kg stronger decline in FFM (β = 0.867; 95%-CI = 0.004–1.730; *p* = 0.049) in men (Figs. 2 and 3, Suppl. Table 1). In women ΔHGS and ΔVO_2_peak were not related to changes in anthropometric markers.

**Fig. 4:**
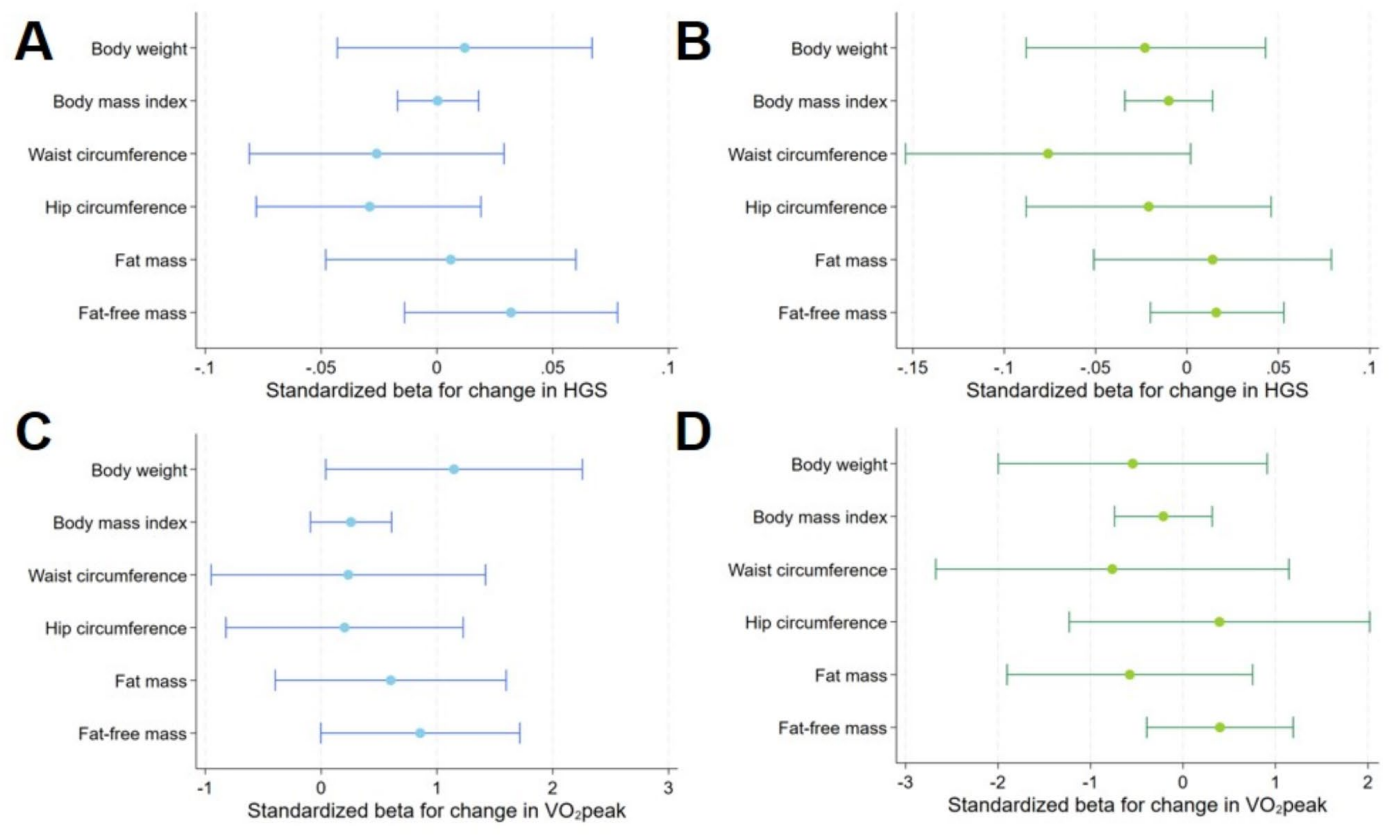
Relationship between change in weight, BMI, waist circumference, hip circumference, fat-mass and fat free mass with hand grip strength and peak oxygen uptake in men (**A**,** C**) and women (**B**,** D**) adjusted for model (2). Model (2) was adjusted for PA, metabolic syndrome, age, baseline outcome, smoking baseline and follow-up. FM is additionally adjusted for FFM and vice versa. Abbreviations: 95%-CI – 95% confidence interval, PA – physical activity, BMI – body mass index, FM – fat mass, FFM – fat free mass, HGS – hand grip strength, VO2peak – peak oxygen uptake.

**Fig. 5:**
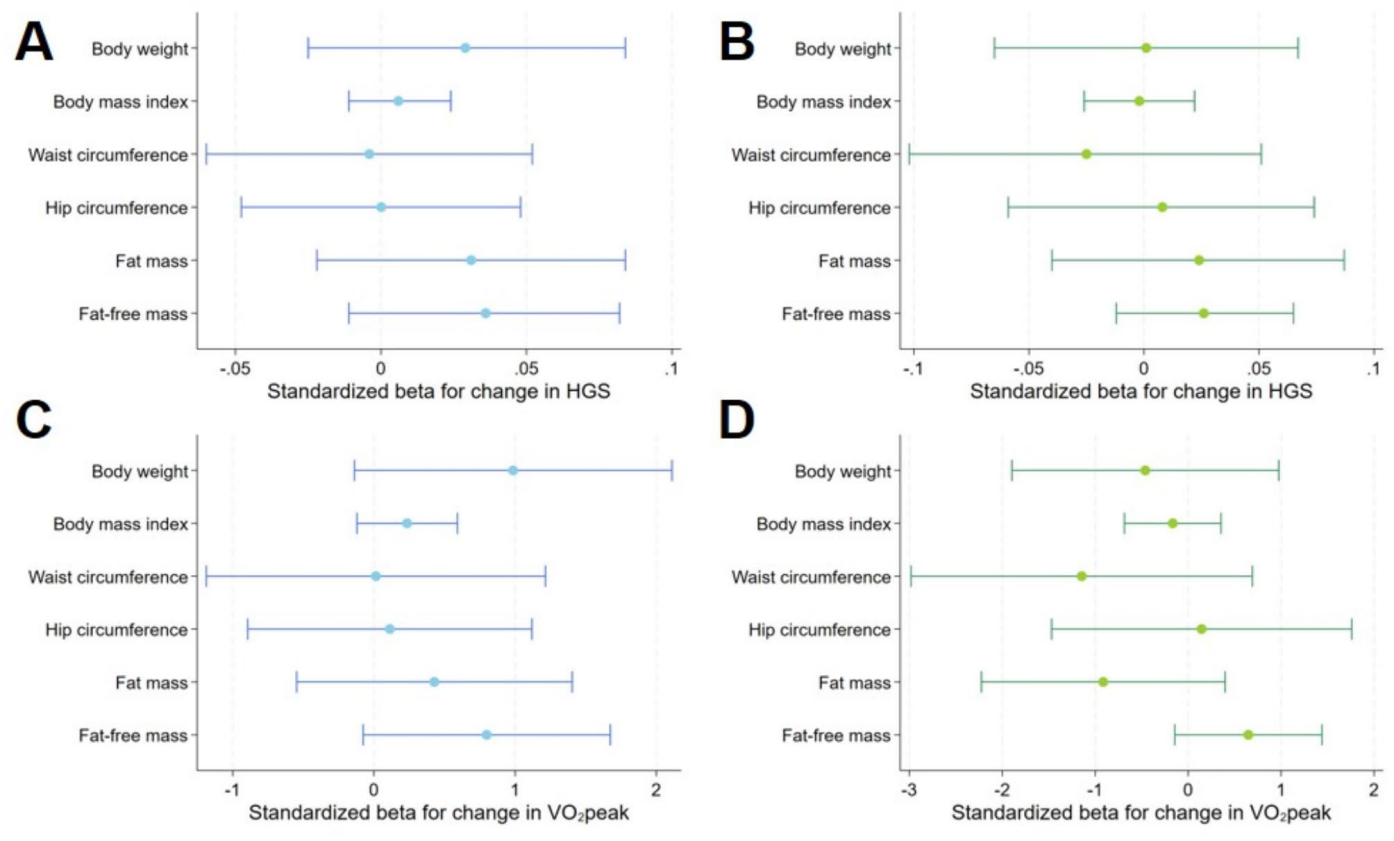
– Relationship between change in weight, BMI, waist circumference, hip circumference, fat-mass and fat free mass with hand grip strength and peak oxygen in men (**A**,** C**) and women (**B**,** D**) uptake adjusted for median outcome instead of baseline. Model (3) was adjusted for median outcome instead of baseline as well as age, smoking status at baseline and follow-up. FM is additionally adjusted for FFM and vice versa. Abbreviations: 95%-CI – 95% confidence interval, BMI – body mass index, FM – fat mass, FFM – fat free mass, HGS – hand grip strength, VO_2_peak – peak oxygen uptake.

Further adjustment for an inactive lifestyle and metabolic syndrome revealed a positive association between ΔVO_2_peak and Δbody weight (Fig. 4, Suppl. Table 2). No further significant associations between ΔHGS or ΔVO_2_peak and Δ body size, shape and composition were detected in men or women. Adjusting the models for the median outcome values instead of baseline outcome values did not alter the results substantially (Fig. 5, Supp. Table 3).

**Fig. 6:**
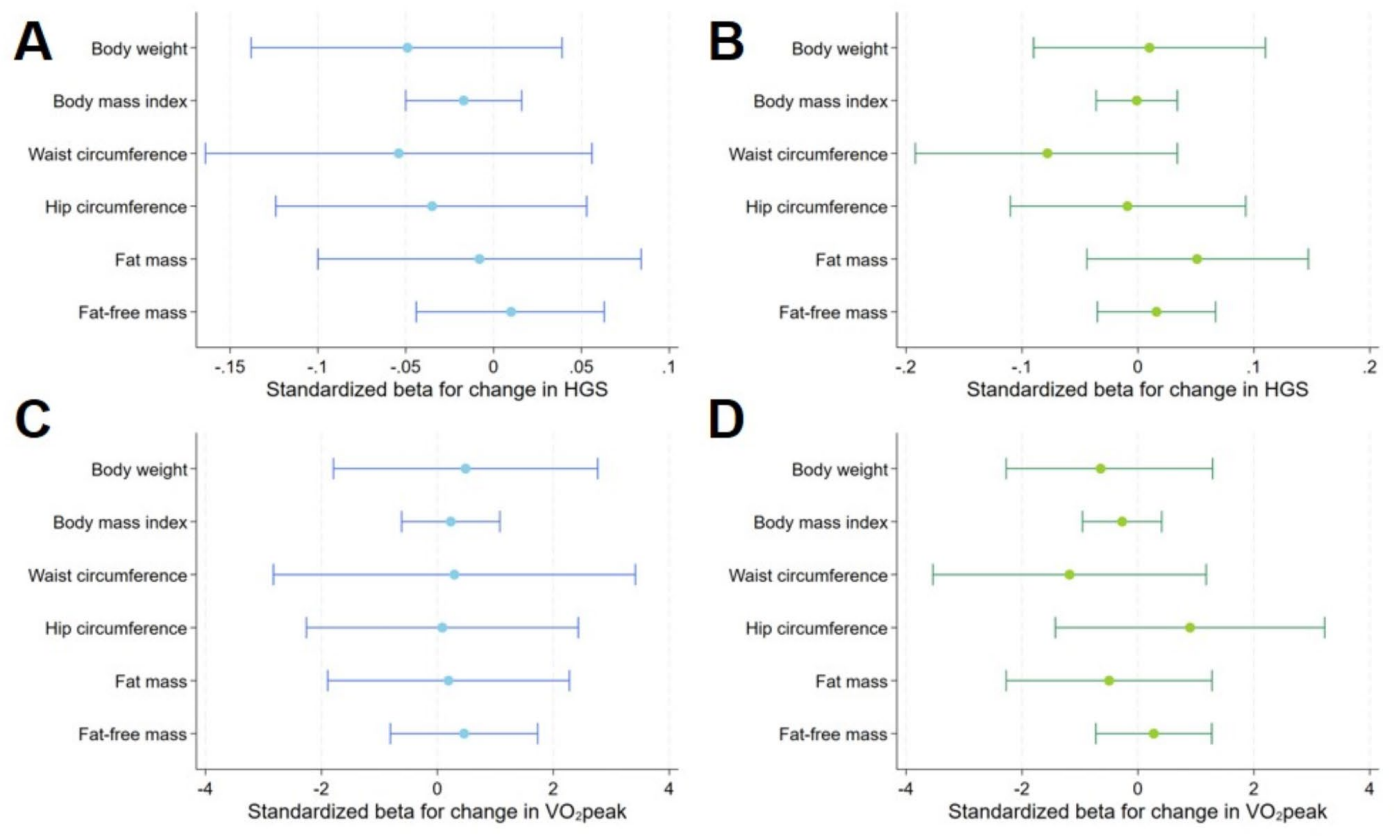
– Relationship between change in weight, BMI, waist circumference, hip circumference, fat-mass and fat free mass with hand grip strength and peak oxygen uptake in pre- (**A**,** C**) and post-menopausal women (**B**,** D**). Abbreviations: 95%-CI – 95% confidence interval, BMI – body mass index, FM – fat mass, FFM – fat free mass, HGS – hand grip strength, VO2peak – peak oxygen uptake.

In order to assess whether the menopausal status may have influenced our results, we stratified the women based on their menopausal status (Fig. 6, Suppl. Table 4). In these models we did not detect any significant association between ΔHGS or ΔVO_2_peak with Δ body size, shape and composition in pre- or post-menopausal women.

Finally, we applied a trajectory analysis for the relationship of ΔHGS or ΔVO_2_peak with changes in body size, shape and composition, in which we could not demonstrate any significant association in men or women (Table 2).

**Table 2:** Trajectory analysis of individuals who either ΔHGS or ΔVO_2_peak.

		5-year-changes in weight		5-year-changes in BMI		5-year-changes in waist circumference	
		Men β (95%-CI)	Women β (95%-CI)	Men β (95%-CI)	Women β (95%-CI)	Men β (95%-CI)	Women β (95%-CI)
5-year-changes in HGS	25th – 75th Percentile	0.018 (-0.531 ; 0.567)	0.289 (-0.364 ; 0.941)	-0.039 (-0.212 ; 0.135)	0.080 (-0.157 ; 0.318)	-0.164 (-0.722 ; 0.395)	-0.009 (-0.783 ; 0.765)
	>= 75th Percentile	0.232 (-0.454 ; 0.918)	-0.073 (-0.797 ; 0.650)	0.032 (-0.185 ; 0.248)	-0.048 (-0.311 ; 0.216)	-0.118 (-0.815 ; 0.580)	-0.618 (-1.475 ; 0.240)
5-year-changes in VO_2_peak	25th- 75th Percentile	0.403 (-0.309 ; 1.115)	0.465 (-0.315 ; 1.244)	0.101 (-0.124 ; 0.326)	0.160 (-0.123 ; 0.444)	0.139 (-0.622 ; 0.900)	0.854 (-0.157 ; 1.864)
	>= 75th Percentile	0.648 (-0.186 ; 1.481)	0.249 (-0.633 ; 1.131)	0.172 (-0.091 ; 0.436)	0.092 (-0.229 ; 0.413)	0.105 (-0.788 ; 0.998)	-0.175 (1.319 ; 0.969)

		5-year-changes in hip circumference		5-year-changes in FM		5-year-changes in FFM	
		Men β (95%-CI)	Women β (95%-CI)	Men β (95%-CI)	Women β (95%-CI)	Men β (95%-CI)	Women β (95%-CI)
5-year-changes in HGS	25th – 75th Percentile	-0.130 (-0.623 ; 0.362)	0.219 (-0.437 ; 0.875)	0.121 (-0.422 ; 0.664)	0.038 (-0.591 ; 0.667)	0.335 (-0.122 ; 0.792)	0.212 (-0.139 ; 0.562)
	>= 75th Percentile	-0.213 (-0.828 ; 0.402)	0.085 (-0.644 ; 0.813)	0.130 (-0.544 ; 0.803)	0.102 (-0.605 ; 0.808)	0.387 (-0.180 ; 0.954)	0.257 (-0.137 ; 0.650)
5-year-changes in VO_2_peak	25th- 75th Percentile	0.185 (-0.473 ; 0.843)	0.339 (-0.527 ; 1.205)	0.218 (-0.415 ; 0.850)	0.215 (-0.495 ; 0.925)	0.425 (-0.118 ; 0.968)	0.151 (-0.266 ; 0.568)
	>= 75th Percentile	0.131 (-0.638 ; 0.900)	0.294 (-0.687 ; 1.274)	0.248 (-0.485 ; 0.982)	0.073 (-0.735 ; 0.881)	0.507 (-0.122 ; 1.137)	0.347 (-0.126 ; 0.821)

The study participants were divided into three groups based on their change from the baseline measurement and to assess the association between ΔHGS or ΔVO_2_peak with anthropometric outcomes. FM is additionally adjusted for FFM and vice versa.

## Discussion

### Summary of findings

HGS, CRF, and body size, shape as well as composition, are important health parameters. The aim of this study was to investigate whether age related changes in CRF and HGS are associated with changes in body size, shape and composition. HGS decreased in men and women, but this change was not related to body size, shape or composition. This is partly in contrast to previous studies. In a cross-sectional study in patients with chronic obstructive pulmonary disease HGS was related to subcutaneous adipose tissue, but not BMI^11^. In 2,339 Brazilian adolescents HGS was inversely associated with fat mass index and positively with lean body mass^12^. In 500 adult Malaysian men and women HGS was associated with BMI^13^. In the 1970 British Cohort Study BMI at ages 10, 16, 30, and 46 years was positively related to HGS at age 46^28^. In elderly participants of the Newcastle 85 + study the decline in HGS was related to FFM independent of sex^29^. Overall, previous research indicated that the relation between HGS and body size, shape and composition is incompletely understood but may depend on age.

Absolute VO_2_peak decreased in men but increased in women during the follow-up. In men the reduction in VO_2_peak was associated with a larger weight gain and lesser FFM. There was no relationship for ΔVO_2_peak and other markers of body size, shape and composition. Interestingly, the observed increase in absolute VO2peak in women was not related to changes in body size, shape or composition despite a significant increase in waist circumference and fat mass during follow-up. In agreement with our findings, in 86 men between the ages of 40 and 72 years the age related decline in VO_2_peak was related to habitual PA but not body weight or composition^30^. In endurance trained women a decline in VO2peak over a seven year period was also not related to body mass^31^. However, in the Aerobics Center Longitudinal Study maintaining a low BMI was related to higher VO_2_peak in 3,429 women and 16,889 men^32^. In summary, age-related declines in physical fitness may be related to PA and anthropometric markers but also depending on age.

One may argue that different lifestyle behaviours influenced our exposure and outcome. Regular physical training increases HGS and CRF^33,34^. In contrast, body size, shape and composition are more strongly influenced by dietary behaviour^35^. The lack of an association between ΔCRF or ΔHGS and body size, shape and composition in our study, possibly supports the argument that physical training changes HGS and CRF while diet influences body size, shape and composition parameters. These results require further investigation in follow-up studies with sex-stratified analysis and information on factors influencing HGS or CRF and their relationship with anthropometrics.

### No relationship between HGS or CRF and body size, shape and composition

A total of 180 Indian students were stratified into low, normal or high BMI. Male students with low and high BMI had lower HGS compared to study participants with normal BMI values^36^. A study with elderly Asian adults showed that total and local muscle mass decline correlated with HGS. This study also found sex-specific effects, since the correlation was weaker in women compared to men^37^. We found no significant correlation between ΔHGS and ΔFFM. However, we only included total muscle mass but not local muscle mass. Additional studies are warranted to differentiate between different muscle beds and types with regards to the association to HGS.

Previous cross-sectional studies also reported no significant relations between CRF with BMI or hip circumference^38^. Although, these studies normalized VO_2_peak to body weight, which may lead to misinterpretation in obese individuals since FFM is responsible for oxygen uptake at a tissue level. For these reasons, standardization to FFM is already recommended^39^. A potential reason for the difference between ours and previous findings is the heterogeneity with regards to the method used to measure body composition (e.g. BIA, DEXA, air displacement plethysmography and imaging techniques)^11,12,14,15^. BIA is a safe, observer-independent, inexpensive and easy-to-perform method. Another potential reason for differences between ours and previous findings are differences in the study populations (e.g. COPD patients, postmenopausal women, elderly etc.)^11,14,16^.

### Sex differences in body composition and fitness

HGS and CRF are significantly higher in men compared to women. In addition, men’s and women’s bodies show sex specific differences in body composition and fitness^10,40,41^. Body shape and composition as well as sex influence the tissue-specific oxygen demand^39^. Women have a higher percentage of total FM, while men have a higher percentage of lean and muscle mass^10^. In addition, in a large population-based survey from the United States FM and obesity-associated genes displayed sex differences for Caucasian study participants^42^. Our results also show a lower FFM and a higher FM in women compared to men at baseline and follow-up.

VO_2_peak is higher in men compared to women. The differences in VO_2_peak between the sexes are attributed to the higher fat percentage, lower muscle mass and lower haemoglobin level in women^43^. In addition, other factors such as sex-specific differences in cardiac filling and performance are important for the differences in VO_2_peak between men and women^41^. Hormonal differences are also central for the sex-specific differences in VO_2_peak. Oestrogen has a favourable effect on oxygen consumption^44^. Therefore, one should consider the hormonal influence of menopause. A previous study already investigated the influence of CRF and FM on oestrogen levels and associated diseases such as breast cancer^16^. After menopause, FM is involved in oestrogen production^45^. Conversely, higher CRF and PA are associated with lower levels of circulating oestrogen^46^. The fact that both CRF and the proportion of FM affect oestrogen levels may explain why menopause did not affect our main results.

### Changes in HGS and CRF with age

In addition to the influence of sex, age is also an important modulator of HGS and CRF. Particularly in older individuals, sarcopenia goes in hand with muscle weakness. HGS may be used to assess vitality, especially in the ageing process^3^. Several studies indicated that HGS decreases with increasing age in both sexes due to the decrease in muscle mass and muscle strength^1,13,37,47^. In our study population, HGS and FFM also decreased in both sexes with increasing age. Previous studies show that VO_2_peak also decreases with age^34,48,49^.Interestingly, in our longitudinal study VO_2_peak decreased only in men. The reasons for the decrease in VO_2_peak in old age are both central (e.g. cardiovascular) and peripheral (e.g. oxygen extraction) physiological mechanisms. Ageing decreases maximum stroke volume, heart rate and the arterio-venous O_2_ difference^48^. Previous studies exploring the decline of CRF with age contradict our results in women, in whom we found an increase in VO_2_peak although PA decreased and metabolic syndrome and anthropometric parameters increased. The reason for this is currently unclear.

### Study strength and limitations

Our study has some limitations. First, we used data from a Caucasian cohort recruiting in a sparsely populated region in eastern Germany. Hence, extending our results to other ethnic groups is not possible. Second, we are unable to track which study participants dropped out between the baseline and follow-up examinations and for what reasons, which is why our results are potentially not representative for the general population. Third, our study is limited to one follow-up. Hence, longer follow-up studies on the relation between CRF or HGS with anthropometric markers are essential. Fourth, the measurements of CRF on the cycle ergometer lead to lower results than on the treadmill^50^. The selection bias in our study is driven by a healthy volunteer bias (i.e. in general individuals who are interested in their health participate in population-based cohort studies). An additional selection bias was induced since the CPET examination takes place on another data and some individuals only participate in the first examination but not the second day.

Our study also has several strengths. The sample under consideration is large (2,496 study participants for HGS and 1,232 study participants for CRF) and covers a wide age range (24–85 years). In addition, well-standardized methods were used for data collection and analysis in both cohorts, which reduces the risk of methodological bias. While multivariable regression models have a risk of falsification, we used a directed acyclic graph to identify appropriate confounders for our analysis (Supplementary Fig. 1 and Supplementary Fig. 2). This does not rule of the possibility of unknown confounding. We also used several different confounders sets which all yielded very similar results.

### Clinical implications of our findings

HGS and CRF are markers for physical strength and fitness. Body size, shape and composition may be used to identify individuals who are overweight or with obesity. Our findings highlight that age-related changes in these parameters are only sparsely related in men and not at all in women. Since strength and fitness may be improved using exercise interventions, these lifestyle changes should be implemented early in life to lessen the age-related decline in HGS and CRF. Further, our findings highlight that exercise interventions are unlikely to result in weight loss, which has been reported previously^51^.

### Future direction

Future studies should explore which factors that change with age are related to changes in anthropometric markers to identify individuals with overweight or obesity in the future. Since our results indicate that markers of physical fitness are not well related with changes in anthropometrics. The positive relationship between anthropometrics and an increased cardiometabolic disease risk is well known. Hence, one may suggest to explore nutritional aspects for changes in anthropometric markers throughout life. Specifically, the role of processed foods over the lifespan should be investigated, given the adverse relation between processed food intake and cardiometabolic disease^52,53^.

## Conclusion

In this large longitudinal study, we report that changes in CRF are related with changes in FFM and body weight in men. We found no relationship between the changes in CRF with anthropometrics in women. Likewise, no correlation was found between the changes in HGS and anthropometric markers in both sexes. We conclude that a possible explanation for our findings is that physical exercise strongly influences CRF and HGS while nutrition has stronger effects on anthropometrics.

## Electronic supplementary material

Below is the link to the electronic supplementary material.

Supplementary Material 1.

Supplementary Material 2.

## Data Availability

The datasets used and/or analysed during the current study available from the corresponding author on reasonable request.

## Abbreviations

95%-CI: 95% confidence interval
BMI: Body mass index
CRF: Cardiorespiratory fitness
CT: Computer tomography
CVD: Cardiovascular disease
DEXA: Dual-energy x-ray absorptiometry
FFM: Fat free mass
FM: Fat mass
HGS: Hand grip strength
PA: Physical activity
SHIP: Study of health in Pomerania
VO_2_peak: Peak oxygen uptake
