# Supplementary material for: The relationship between age related changes in strength and fitness with body size, shape and composition
Source: Sci Rep. 2025 Mar 21;15:9833. doi: 10.1038/s41598-025-93828-2 (PMC11928602; doi:10.1038/s41598-025-93828-2)
Supplement: Supplementary file 2 — Supplementary Material 2. [file 41598_2025_93828_MOESM2_ESM.pdf]

Supplementary material for

The relationship between age related changes in strength and fitness with anthropometric markers

by Schulte et al.

Supplementary Figure 1

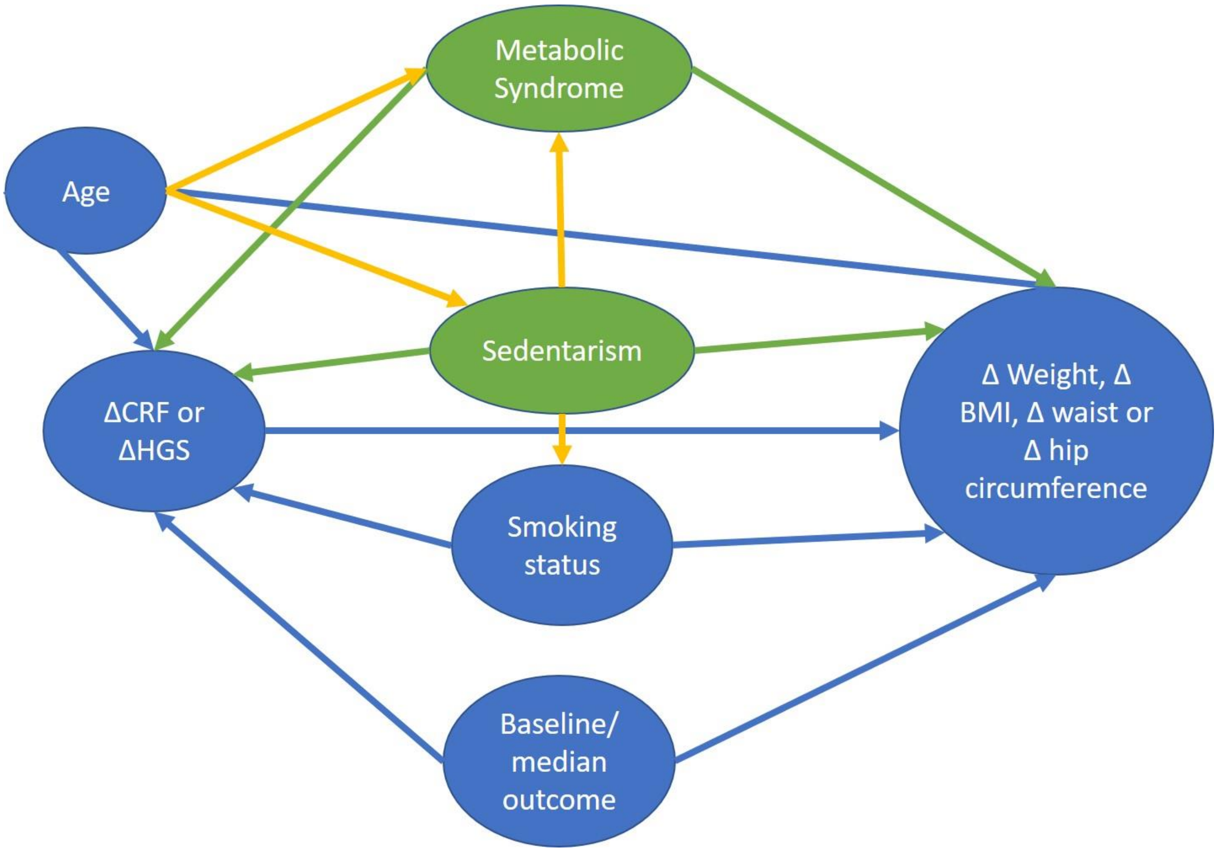

Supplementary Figure 1 – Directed acyclic graph for the relationship between ΔCRF or ΔHGS with Δweight, body mass index, hip or waist circumference, abbreviations: BMI – body mass index, CRF – cardio-respiratory fitness, HGS – hand grip strength

Supplementary Figure 2

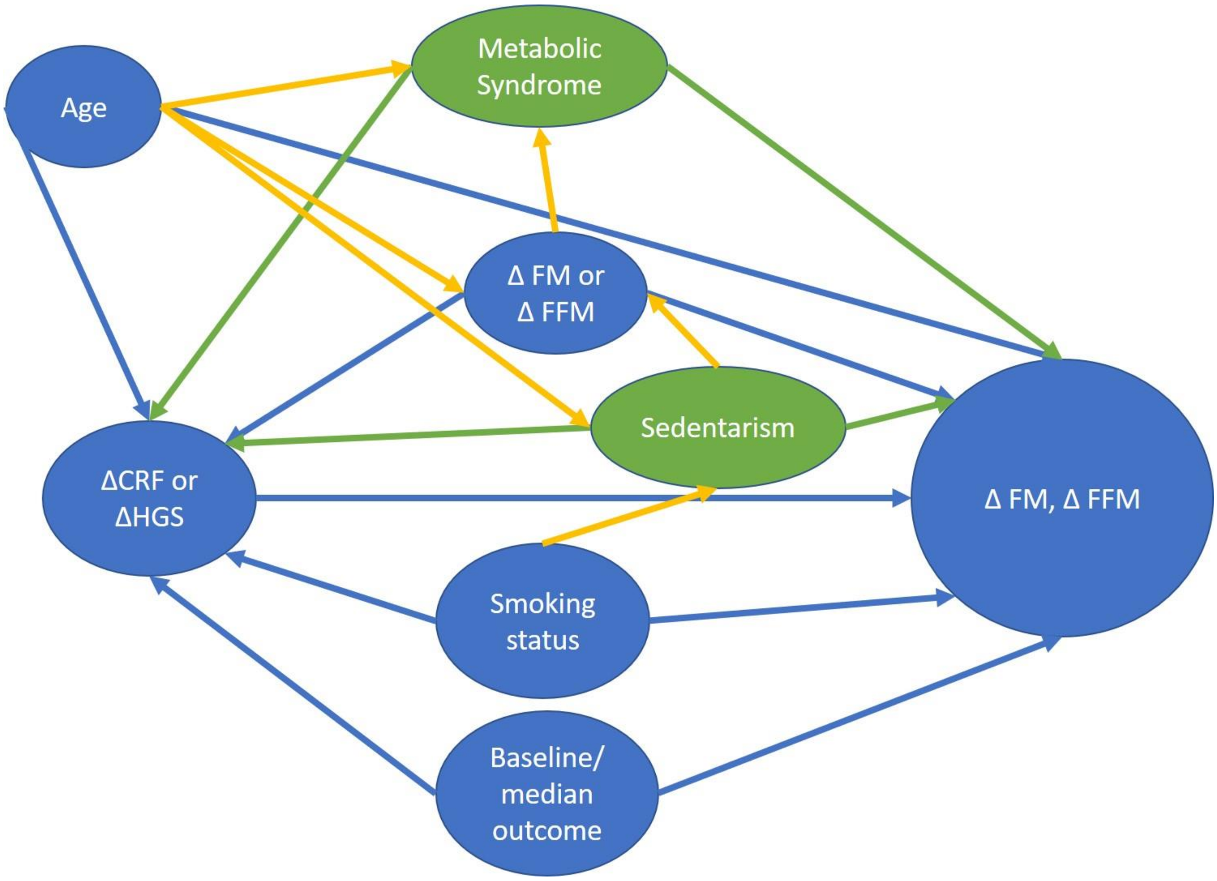

Supplementary Figure 2 – Directed acyclic graph for the relationship between ΔCRF or ΔHGS with ΔFM or FFM. Abbreviations: BMI – body mass index, CRF – cardio-respiratory fitness, FM – fat mass, FFM – fat free mass, HGS – hand grip strength

Suppl. Table 1: Relationship between change in weight, BMI, waist circumference, hip circumference, fat-mass and fat free mass with hand grip strength and peak oxygen uptake

	5-year-changes in weight		5-year-changes in BMI		5-year-changes in waist circumference	
	Men β (95%-CI)	Women β (95%-CI)	Men β (95%-CI)	Women β (95%-CI)	Men β (95%-CI)	Women β (95%-CI)
5-year-changes in HGS	0.008 (-0.047 ; 0.064)	-0.025 (-0.092; 0.041)	-0.001 (-0.019 ; 0.016)	-0.011 (-0.035 ; 0.013)	-0.030 (-0.087 ; 0.026)	-0.072 (-0.152 ; 0.007)
5-year-changes in VO_2peak	1.101 (-0.035 ; 2.237)	-0.192 (-1.656 ; 1.272)	0.245 (-0.114 ; 0.605)	-0.070 (-0.603 ; 0.463)	0.163 (-1.055 ; 1.380)	-0.606 (-2.515 ; 1.302)

	5-year-changes in hip circumference		5-year-changes in FM		5-year-changes in FFM	
	Men β (95%-CI)	Women β (95%-CI)	Men β (95%-CI)	Women β (95%-CI)	Men β (95%-CI)	Women β (95%-CI)
5-year-changes in HGS	-0.029 (-0.079 ; 0.021)	-0.027 (-0.094 ; 0.040)	-0.0003 (-0.055 ; 0.054)	0.015 (-0.051 ; 0.081)	0.027 (-0.019 ; 0.073)	0.013 (-0.024 ; 0.050)
5-year-changes in VO_2peak	0.159 (-0.891 ; 1.209)	0.475 (-1.149; 2.099)	0.606 (-0.400 ; 1.613)	-0.255 (-1.589; 1.079)	**0.867 (0.004; 1.730)**	0.421 (-0.363 ; 1.205)

Model (1) was adjusted for age, baseline outcome, smoking baseline and follow-up. FM is additionally adjusted for FFM and vice versa. Abbreviations: BMI – body mass index, FM – fat mass, FFM – fat free mass, HGS – hand grip strength, VO_2peak – peak oxygen uptake

Suppl. Table 2: Relationship between change in weight, BMI, waist circumference, hip circumference, fat-mass and fat free mass with hand grip strength and peak oxygen uptake adjusted for model (2).

	5-year-changes in weight		5-year-changes in BMI		5-year-changes in waist circumference	
	Men<br>β (95%-CI)	Women<br>β (95%-CI)	Men<br>β (95%-CI)	Women<br>β (95%-CI)	Men<br>β (95%-CI)	Women<br>β (95%-CI)
5-year-changes in HGS	0.012<br>(-0.043 ; 0.067)	-0.023<br>(-0.088 ; 0.043)	0.0003<br>(-0.017 ; 0.018)	-0.010<br>(-0.034 ; 0.014)	-0.026<br>(-0.081 ; 0.029)	-0.076<br>(-0.154 ; 0.002)
5-year-changes in VO_2peak	**1.148**<br>**(0.040 ; 2.256)**	-0.543<br>(-1.997 ; 0.911)	0.258<br>(-0.092 ; 0.609)	-0.212<br>(-0.740 ; 0.316)	0.235<br>(-0.949 ; 1.420)	-0.764<br>(-2.673 ; 1.146)
	**5-year-changes in hip circumference**		**5-year-changes in FM**		**5-year-changes in FFM**	
	Men<br>β (95%-CI)	Women<br>β (95%-CI)	Men<br>β (95%-CI)	Women<br>β (95%-CI)	Men<br>β (95%-CI)	Women<br>β (95%-CI)
5-year-changes in HGS	-0.029<br>(-0.078 ; 0.019)	-0.021<br>(-0.088 ; 0.046)	0.006<br>(-0.048 ; 0.060)	0.014<br>(-0.051 ; 0.079)	0.032<br>(-0.014 ; 0.078)	0.016<br>(-0.020 ; 0.053)
5-year-changes in VO_2peak	0.203<br>(-0.822 ; 1.227)	0.395<br>(-1.230 ; 2.020)	0.601<br>(-0.396 ; 1.598)	-0.575<br>(-1.903 ; 0.753)	0.856<br>(-0.003 ; 1.716)	0.400<br>(-0.391 ; 1.192)

Model (2) was adjusted for PA, metabolic syndrome, age, baseline outcome, smoking baseline and follow-up. FM is additionally adjusted for FFM and vice versa. Abbreviations: 95%-CI – 95% confidence interval, PA – physical activity, BMI – body mass index, FM – fat mass, FFM – fat free mass, HGS – hand grip strength, VO_2peak – peak oxygen uptake

Suppl. Table 3: Relationship between change in weight, BMI, waist circumference, hip circumference, fat-mass and fat free mass with hand grip strength and peak oxygen uptake adjusted for median outcome instead of baseline.

	5-year-changes in weight		5-year-changes in BMI		5-year-changes in waist circumference	
	Men β (95%-CI)	Women β (95%-CI)	Men β (95%-CI)	Women β (95%-CI)	Men β (95%-CI)	Women β (95%-CI)
5-year-changes in HGS	0.029 (-0.025 ; 0.084)	0.0008 (-0.065 ; 0.067)	0.006 (-0.011 ; 0.024)	-0.002 (-0.026 ; 0.022)	-0.004 (-0.060 ; 0.052)	-0.025 (-0.102 ; 0.051)
5-year-changes in VO_2peak	0.986 (-0.138 ; 2.111)	-0.461 (-1.896 ; 0.975)	0.235 (-0.120 ; 0.591)	-0.166 (-0.686 ; 0.354)	0.014 (-1.187 ; 1.215)	-1.145 (-2.981 ; 0.691)

	5-year-changes in hip circumference		5-year-changes in FM		5-year-changes in FFM	
	Men β (95%-CI)	Women β (95%-CI)	Men β (95%-CI)	Women β (95%-CI)	Men β (95%-CI)	Women β (95%-CI)
5-year-changes in HGS	0.0001 (-0.048 ; 0.048)	0.008 (-0.059 ; 0.074)	0.031 (-0.022 ; 0.084)	0.024 (-0.040 ; 0.087)	0.036 (-0.011 ; 0.082)	0.026 (-0.012 ; 0.065)
5-year-changes in VO_2peak	0.113 (-0.894 ; 1.119)	0.145 (-1.469 ; 1.759)	0.428 (-0.547 ; 1.404)	-0.913 (-2.225 ; 0.398)	0.799 (-0.076 ; 1.674)	0.648 (-0.144 ; 1.440)

Model (3) was adjusted for median outcome instead of baseline as well as age, smoking status at baseline and follow-up. FM is additionally adjusted for FFM and vice versa. Abbreviations: 95%-CI – 95% confidence interval, BMI – body mass index, FM – fat mass, FFM – fat free mass, HGS – hand grip strength, VO_2peak – peak oxygen uptake

Supp. Table 4: Relationship between change in weight, BMI, waist circumference, hip circumference, fat-mass and fat free mass with hand grip strength and peak oxygen uptake stratified by menopause status.

		5-year-changes in weight β (95%-CI)	5-year-changes in BMI β (95%-CI)	5-year-changes in waist circumference β (95%-CI)
5-year-changes in HGS	Menopause No	-0.049 (-0.138 ; 0.039)	-0.017 (-0.050 ; 0.016)	-0.054 (-0.164 ; 0.056)
	Menopause Yes	0.010 (-0.090 ; 0.110)	-0.001 (-0.036 ; 0.034)	-0.078 (-0.192 ; 0.034)
5-year-changes in VO_2peak	Menopause No	0.487 (-1.792 ; 2.766)	0.231 (-0.616 ; 1.078)	0.294 (-2.828 ; 3.416)
	Menopause Yes	-0.643 (-2.272 ; 1.288)	-0.272 (-0.956 ; 0.411)	-1.179 (-3.536 ; 1.177)
		5-year-changes in hip circumference β (95%-CI)	5-year-changes in FM β (95%-CI)	5-year-changes in FFM β (95%-CI)
5-year-changes in HGS	Menopause No	-0.035 (-0.124 ; 0.053)	-0.008 (-0.100 ; 0.084)	0.010 (-0.044 ; 0.063)
	Menopause Yes	-0.009 (-0.110 ; 0.093)	0.051 (-0.044 ; 0.147)	0.016 (-0.035 ; 0.067)
5-year-changes in VO_2peak	Menopause No	0.086 (-2.259 ; 2.432)	0.193 (-1.891 ; 2.278)	0.461 (-0.810 ; 1.731)
	Menopause Yes	0.899 (-1.424 ; 3.222)	-0.497 (-2.272 ; 1.278)	0.274 (-0.726 ; 1.274)

Abbreviations: 95%-CI – 95% confidence interval, BMI – body mass index, FM – fat mass, FFM – fat free mass, HGS – hand grip strength, VO_2peak – peak oxygen uptake

Suppl. Table 5: R^2 for the relationship between change in weight, BMI, waist circumference, hip circumference, fat-mass and fat free mass with hand grip strength and peak oxygen uptake

	5-year-changes in weight		5-year-changes in BMI		5-year-changes in waist circumference	
	Men R^2	Women R^2	Men R^2	Women R^2	Men R^2	Women R^2
5-year-changes in HGS	0.1096	0.0779	0.0800	0.0520	0.0338	0.0169
5-year-changes in VO_2peak	0.1114	0.0790	0.0821	0.0523	0.0338	0.0119
	5-year-changes in hip circumference		5-year-changes in FM		5-year-changes in FFM	
	Men R^2	Women R^2	Men R^2	Women R^2	Men R^2	Women R^2
5-year-changes in HGS	0.0198	0.0389	0.1467	0.0780	0.2047	0.1251
5-year-changes in VO_2peak	0.0225	0.0247	0.1804	0.0936	0.2265	0.0930

Model (1) was adjusted for age, baseline outcome, smoking baseline and follow-up. FM is additionally adjusted for FFM and vice versa. Abbreviations: BMI – body mass index, FM – fat mass, FFM – fat free mass, HGS – hand grip strength, VO_2peak – peak oxygen uptake
